# Modeling activation of inflammatory response system: a molecular-genetic neural network analysis

**DOI:** 10.1186/1753-6561-1-s1-s61

**Published:** 2007-12-18

**Authors:** Hans H Stassen, Armin Szegedi, Christian Scharfetter

**Affiliations:** 1 Psychiatric University Hospital, P.O. Box 1931, CH-8032 Zurich, Switzerland; 2 Global Clinical Development, Organon International, 56 Livingston Avenue, Roseland, New Jersey 07068, USA

## Abstract

Significant alterations of T-cell function, along with activation of the inflammatory response system, appear to be linked not only to treatment-resistant schizophrenia, but also to functional psychoses and mood disorders. Because there is a relatively high comorbidity between rheumatoid arthritis (RA), schizophrenia and major depression, the question arises whether there is a common, genetically modulated inflammatory process involved in these disorders. On the basis of three family studies from the U.S. and Europe which were ascertained through an index case suffering from RA (599 nuclear families, 1868 subjects), we aimed to predict the inter-individual variation of autoantibody IgM levels, as an unspecific indicator of inflammatory processes, through molecular-genetic factors. In a three-stage strategy, we first used nonparametric linkage (NPL) analysis to construct an initial configuration of genomic loci showing a sufficiently high NPL score in all three populations. This initial configuration was then modified by iteratively adding or removing genomic loci such that genotype-phenotype correlations were improved. Finally, neural network analysis (NNA) was applied to derive classifiers that predicted the phenotype from the multidimensional genotype. Our analysis led to an activation model that predicted individual IgM levels from the subjects' multidimensional genotypes very reliably. This allowed us to use the activation model for an analysis of the DNA of an existing sample of 1003 psychiatric patients in order to test, in a first approach, whether a deviant, genetically modulated inflammatory process is involved in the pathogenesis of major psychiatric disorders.

## Background

While the ultimate goal of molecular-genetic research is the detection of causality, clinicians are also interested in reliable classification and prediction through objective laboratory methods. Classification and prediction do not require full understanding of causality but can, nonetheless, contribute to significantly improved treatments. This is particularly true for rheumatoid arthritis (RA), where autoantibody formation develops years before the first symptoms of RA occur. From the psychiatric point of view, it is most intriguing that active immune processes may be involved in the pathogenesis of major psychiatric disorders, as suggested by evidence from recent studies. Specifically, significant alterations of T-cell function, along with activation of the inflammatory response system, appear to be linked to treatment-resistant schizophrenia [1]. Similar processes have also been reported for mood disorders in general [2]. The abnormalities of central nervous system (CNS) metabolism observed with functional psychoses and depression might, therefore, arise because genetically modulated inflammatory reactions damage the microvascular system of the brain, with the nature of the infectious agent being less important than the patients' genetically influenced inflammatory response [3]. Rheumatoid factor IgM is in use as a diagnostic test for RA, but possesses a low specificity [4]. In particular, because IgM autoantibody formation develops years before the first symptoms of RA occur [5], IgM levels may well be related to the patients' genetically predisposed inflammatory response system, and may even be related to autoimmune diseases in general. The pathogenesis of these diseases, however, is insufficiently understood, also because the question of autoantibody appearance prior to inflammation – indicating an antibody-driven inflammatory response – has not yet been answered on the basis of empirical data.

## Methods

### Neural network analysis (NNA)

NNA provides powerful tools for modeling pre-specified responses to complex, multidimensional input stimuli. It is the specific advantage of NNA that no causal relationship between stimuli and response is required. NNA connects the "neurons" of input and output layers via one or more "hidden" layers. All outputs are computed using sigmoid thresholding of the scalar product of the corresponding weight and input vectors. All outputs at stage "s" are connected to each input of stage "s + 1". The most popular learning strategy, the back-propagation algorithm, looks for the minimum of the error function in the weight space (goodness of fit) using the method of gradient descent. The basic algorithm is:

(i) output:   $s_{i}=\sigma \left[\sum_{j}w_{ij}s_{j}\right]$   *y*_i _observed   (*i *= 1, 2,...,*N*_i_)

(j) hidden layer:   $s_{j}=\sigma \left[\sum_{k}w_{jk}s_{k}\right]$   (*j *= 1, 2,...,*N*_j_)

(k) input:   *s*_*k *_= *x*_*k*_   *x*_*k *_observed   (*k *= 1, 2,...,*N*_k_)

   improvements:   $\begin{array}{cc} \Delta w_{ij}=\alpha \cdot \varepsilon_{i}^{\nu }\cdot s_{j}\cdot s_{i}(1-s_{i}) & \varepsilon_{i}^{\nu }=y_{i}^{\nu }-s_{i}^{\nu } \end{array}$   (*ν *= 1, 2,..,*p*)

$$\Delta w_{jk}=\alpha \cdot \sum_{i=1}^{N_{i}}\varepsilon_{i}^{\nu }\cdot s_{k}\cdot s_{i}(1-s_{i})\cdot w_{ij}\cdot s_{j}(1-s_{j}),$$

where *x*_*k *_denotes observed stimuli, *y*_*i *_denotes observed responses, *σ *denotes the activation function of sigmoid-type: R → (0, 1), *α *denotes the learning rate, and *p *is the number of probes (genotyped subjects). This algorithm can easily be adapted to genetic models.

### *k*-Fold cross-validation

Results derived through the standard NNA approach, which uses 80% of samples for training and the remaining 20% for testing, tend to be over optimistic, in particular if genotype errors and missing data are present. Therefore, in the *k*-fold cross-validation, the data are split into *k *roughly equal parts, and *k *- 1 partitions are used for training, while one partition is used for testing. The process is repeated until each partition has served as a testing set, so that *k *estimates of prediction error are generated. The choice of *k *is crucial in this approach, because the resulting prediction error is approximately unbiased for the "true" error only for sufficiently large *k *(*k *≈ 10 is a typical value in practice).

### Genetic vector spaces

Once a function is defined that quantifies the genetic distance between any two subjects with *n*-dimensional genotype patterns at *n *loci, the Housholder-Torgerson formula

$$bjk=\frac{1}{2}\left(\frac{1}{n}\sum_{j}^{n}d_{jk}^{2}+\frac{1}{n}\sum_{k}^{n}d_{jk}^{2}-\frac{1}{n^{2}}\sum_{j}^{n}\sum_{k}^{n}d_{jk}^{2}-d_{jk}^{2}\right)$$

gives a routine method for computing directly from the inter-individual genetic distances *d*_*jk *_a matrix (*b*_*jk*_) of scalar products between points with origin at the centroid of all of the points. The matrix is then factored by any of the usual factoring procedures to obtain the projections of the points onto r orthogonal axes of a vector space. In this metric vector space, individuals are characterized as distinct "points" in such a way that individuals with similar genotype patterns form compact clouds, while genetically dissimilar individuals are located in more distant regions. Accordingly, one expects the groupings associated with different IgM classes to be well separated in a "genetic" vector space constructed from those genomic loci that influence IgM levels.

### Learning to recognize: three-stage adaptive strategy

Although the detection of causal genotype-phenotype relationships (in the strict sense) was not the primary goal of our analysis, we have taken special precautions to ensure that a biologically meaningful solution was established. Specifically, we used a three-stage strategy: 1) nonparametric linkage (NPL) analysis across three independently ascertained family samples was applied for initial signal detection; 2) the initial configuration was then modified by iteratively adding or removing genomic loci to increase genotype-phenotype correlations; 3) subsequent NNA was used to weight genomic loci and their interactions optimally. Nonetheless, all of these steps do not necessarily establish biological meaningfulness, but merely identify genomic regions likely to harbor functional DNA polymorphisms that are causally related to the trait of interest. Accordingly, our approach to establishing biological significance also involves a large-scale SNP analysis using 5728 selected SNPs. Because there are complex patterns of linkage disequilibrium and haplotype block structure across the whole genome with strong nonlinearities, special techniques are necessary to narrow in on candidate regions successfully [6]. Results derived from this SNP analysis are not presented.

Our sample comprised 599 nuclear families (NARAC screen 1: 256; NARAC screen 2: 255; France: 88) with 1868 genotyped subjects (718 + 717 + 433) who were genotyped for either 396 (NARAC) or 1083 microsatellites (France). An integrated genetic map was constructed on the basis of deCODE and NCBI-36 data, so that the three populations could be compared through NPL analyses. On the phenotype level, the quantitative clinical measure rheumatoid factor IgM was available for the NARAC screens, whereas the French data included only a dichotomous affected/unaffected measure. For the NPL analyses, which were carried out independently for the three populations under investigation, we relied on the dichotomous measures under the assumption of a sufficiently close association between RA and the measured antibody IgM, while the optimization procedures (NNA, genetic vector space method) evaluated the quantitative measures of NARAC screens 1 and 2 for which we defined three subject classes using IgM levels: 1) normal: 0 ≤ IgM < 13.5, 2) low: 13.5 ≤ IgM < 50, and 3) elevated: 50 ≤ IgM. Due to incomplete data, only 926 subjects could be included.

## Results

**Figure 1:**
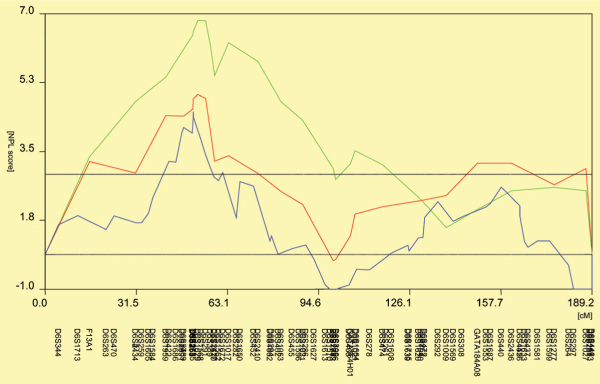
**NPL analysis**. NPL analyses carried out separately for the three populations: NARAC screen1 (green), NARAC screen2 (red), and French samples (blue) yielded several candidate regions which showed significant NPL scores across the three samples under investigation.

**Figure 2:**
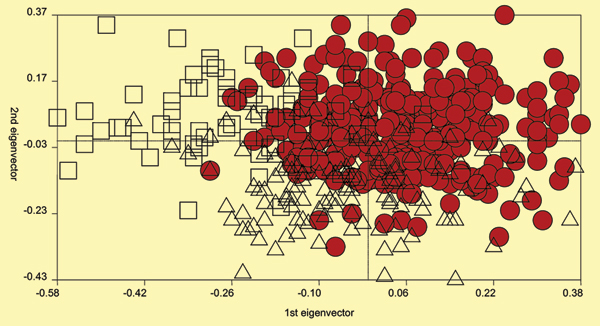
**Projection of 926 subjects onto the plane defined by the two largest eigenvectors of a genetic vector space spanned by 16 polymorphisms**. The projections revealed differences on the genotype level between IgM-related groups: circles, normal subjects (0 ≤ IgM < 13.5; *n *= 576; 100% correctly classified by subsequent NNA); triangles, subjects with low IgM levels (13.5 ≤ IgM < 50; *n *= 237; 77.6% correctly classified by subsequent NNA); squares, subjects with elevated IgM levels (*n *= 113; 98.2% correctly classified by subsequent NNA).

NPL analyses carried out separately for the three populations under investigation revealed several candidate regions that showed significant NPL scores in all three samples (Figure 1). Twenty markers from these candidate regions then served as starting configuration for an iterative optimization of genotype-phenotype correlations: 1) using a set-theoretical similarity measure [7] we computed the 926(926 - 1)/2 genetic similarities *s*_*jk *_between any two subjects *j*, *k*; 2) from these *s*_*jk *_we constructed a genetic vector space; 3) linear discriminant analysis of the three IgM classes then yielded an estimate of the underlying genotype-phenotype correlation; and 4) genotype-phenotype correlations were improved by iteratively adding or removing genomic loci and repeating steps 1 to 3. Results suggested a configuration of 16 polymorphisms on chromosomes 1, 2, 5, 6, 11, and 22 which allowed a fairly powerful classification of subjects by means of linear discriminant analysis, offering an overall performance of 73.8% across NARAC screens 1 and 2. The overlap between the subgroups, as seen in Figure 2, speaks against direct clinical application of these classifiers; therefore, the configuration's predictive power needed boosting through NNA.

Subsequent NNA (subjects with >12.5% missing data removed; 926 probes: 576 normal, 237 low, 113 elevated; 5.8% missing data; three layers; 32 input nodes; 40 nodes on hidden layer; 0.05 convergence criterion; 40,000 iterations; 0.2 learning rate), under the constraint of reproducibility across NARAC screens 1 and 2, yielded weights that enabled re-classification of subjects through genotype-based classifiers at a sensitivity and specificity of >90% (Table 1), thus indicating that the chosen polymorphisms possess an informativeness high enough to enable prediction of correct IgM class for almost all probes under investigation. Given missing data rates of up to 12.5% per subject and an estimated genotype error rate of 5%, it is not really surprising that *k*-fold cross-validation (*k *= 10) revealed reduced overall performances in the range of 78.2% (± 3.9) which, nonetheless, mean slight improvement compared to 73.8% derived through discriminant analysis.

**Table 1:** Genotype-based classification of subjects with respect to IgM levels

IgM level	*N*	Prevalence	Normal	Low	Elevated	Sensitivity	Specificity
Normal	576	62.2%	**576**^a^	0	0	0.988	0.997
Low	237	25.6%	1	**184**	52	0.776	0.987
Elevated	113	12.2%	0	2	**111**	0.982	0.936

Neural Network Analysis yielded weights that enabled re-classification of 926 subjects with respect to IgM levels through genotype-based classifiers at a sensitivity and specificity of >90% (with the exception of 23.4% of low IgM level subjects who were classified as subjects with elevated IgM levels).

^a^Bold text indicates correctly re-classified subjects prior to *k*-fold cross-validation.

## Conclusion

This study has demonstrated the feasibility of deriving sufficiently sensitive and specific genotype-based classifiers through NNA. However, NNA does not necessarily establish a causal relationship between stimuli (input) and responses (output). Epiphenomena that are only indirectly related, or may even be physiologically unrelated, to the inflammatory response system are likely to explain the observed genotype-based, reproducible classification of patient IgM levels. Even though our current activation model with its overall performance of 78.2% does not yet meet the clinical requirements of diagnostic tools, its performance is high enough to justify an analysis of the DNA of our existing sample of 1003 psychiatric patients so that the hypothesis of whether deviant, genetically modulated inflammatory processes are involved in the pathogenesis of major psychiatric disorders can be tested in a first approach.

## Competing interests

The author(s) declare that they have no competing interests.
